# Supplementary figures and images for: PPAR-γ activation promotes xenogenic bioroot regeneration by attenuating the xenograft induced-oxidative stress
Source: Int J Oral Sci. 2023 Feb 16;15:10. doi: 10.1038/s41368-023-00217-4 (PMC9935639; doi:10.1038/s41368-023-00217-4)

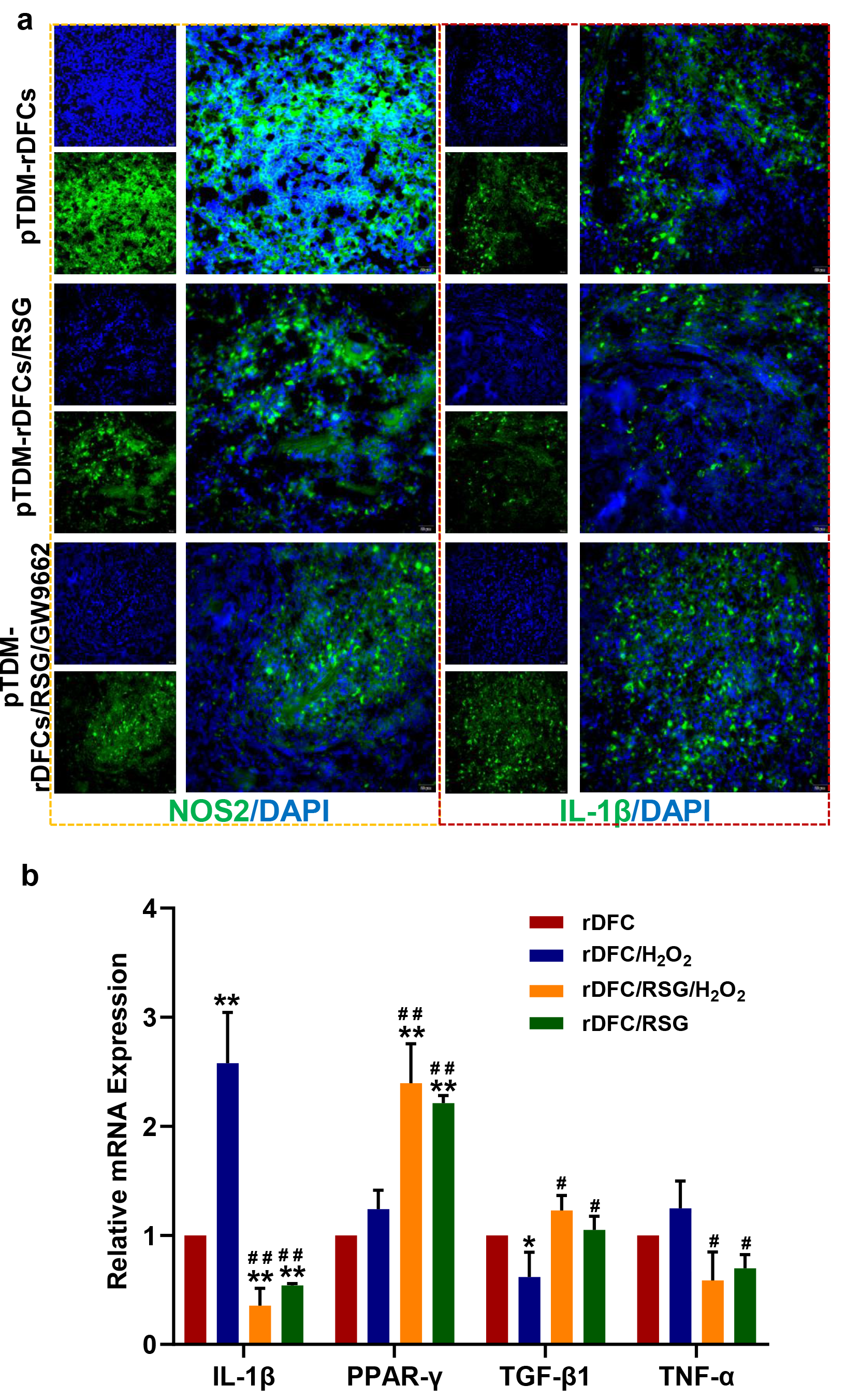

Supplement: Supplementary file 1 — Figure S1 [file 41368_2023_217_MOESM1_ESM.tif]

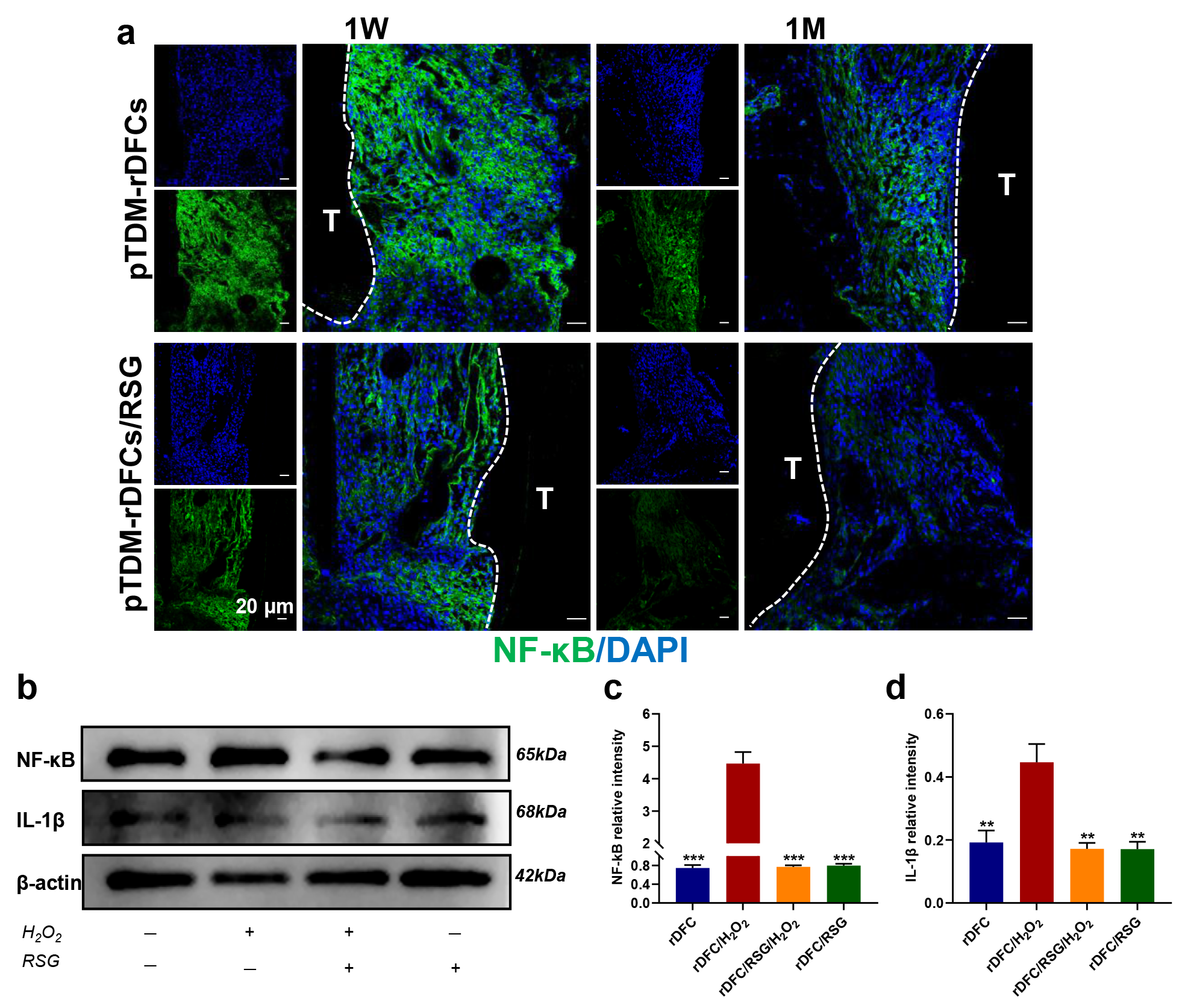

Supplement: Supplementary file 2 — Figure S2 [file 41368_2023_217_MOESM2_ESM.tif]
