# Supplementary material for: PPAR-γ activation promotes xenogenic bioroot regeneration by attenuating the xenograft induced-oxidative stress
Source: Int J Oral Sci. 2023 Feb 16;15:10. doi: 10.1038/s41368-023-00217-4 (PMC9935639; doi:10.1038/s41368-023-00217-4)
Supplement: Supplementary file 3 — Title page [file 41368_2023_217_MOESM3_ESM.docx]

Figure S1. RSG preconditioning could attenuate inflammatory reaction. a, expressions of NOS2 (inducible nitric oxide synthase) and IL-1 β around the xenograft implanted in the mandible of rats at 1 week post surgery. GW9662, an antagonist of PPARγ, was administrated to explore the role of PPARγ in regulating the inflammatory response. Compared to the pTDM-rDFCs group, RSG administration could largely decrease the expression of NOS2 and IL-1 β, while GW9662 could partly reverse this anti-inflammatory effect, confirming the role of PPARγ of RSG in attenuating the inflammatory action. b, mRNA expressions of TGF-β1, IL-1 β, TNF-α and PPARγof cells treated with or without H_2_O_2_ and/or RSG for 4 hours. RSG preconditioning could obviously decrease the relative expression of IL-1 β and TNF-α while increase TGF-β1 level by upregulating PPARγ expression. *p < 0.05, **p < 0.01 versus rDFC; ^#^p < 0.05, ^##^p < 0.01 versus rDFC/H_2_O_2_.

Figure S2. RSG preconditioning could attenuate inflammatory reaction by suppressing NF-κB expression. a, expressions of NF-κB around the xenograft implanted in the mandible of rats at 1 week and 1 month post surgery. Compared to the pTDM-rDFCs group, RSG administration could largely decrease the expression of NF-κB both at 1 week and 1 month, confirming the role of NF-κB of RSG in attenuating the inflammatory action. T: TDM; scale bar=20 μm；b, protein expression of IL-1 β and NF-κB of the cells treated with or without H_2_O_2_ and/or RSG for 4 hours. RSG preconditioning could obviously decrease the relative expression of IL-1 β and NF-κB. c and d, relative expression of NF-κB and IL-1 β. Relative intensity of NF-κB in rDFC/H2O2 group is more than 4 times than that in rDFC group while RSG administration can decrease the NF-κB expression to a normal level; similar to this result, IL-1 β expression in rDFC/H2O2 group is more than 2 times than that in rDFC group while RSG administration can decrease the IL-1 β expression to a normal level. **p < 0.01 versus rDFC/H_2_O_2_, ***p < 0.001 versus rDFC/H_2_O_2_
